# When Low Leisure-Time Physical Activity Meets Unsatisfied Psychological Needs: Insights From a Stress-Buffer Perspective

**DOI:** 10.3389/fpsyg.2018.02097

**Published:** 2018-11-02

**Authors:** Markus Gerber, Sandrine Isoard-Gautheur, René Schilling, Sebastian Ludyga, Serge Brand, Flora Colledge

**Affiliations:** ^1^Department of Sport, Exercise and Health, Sport Science Section, University of Basel, Basel, Switzerland; ^2^Laboratoire Sport et Environnement Social, Université Grenoble Alpes, Grenoble, France; ^3^Center for Affective, Stress and Sleep Disorders, University of Basel, Basel, Switzerland; ^4^Substance Abuse Prevention and Sleep Disorders Research Center, Kermanshah University of Medical Sciences, Kermanshah, Iran

**Keywords:** autonomy, burnout, competence, relatedness, self-determination theory, stress

## Abstract

**Background:** Few studies have tested whether the stress-buffering effects of leisure-time physical activity (LTPA) depend on other resources, such as the satisfaction of basic psychological needs. Therefore, the present study examines the interaction between perceived stress, LTPA and psychological need satisfaction (PNS) on occupational burnout symptoms in a sample of Swiss workers.

**Methods:** The sample consisted of 306 employees (48% women; *M*_age_ = 42.9 years, *SD* = 14.1). Perceived stress was assessed with the Perceived Stress Scale, LTPA with the International Physical Activity Questionnaire, PNS (autonomy, relatedness, and competence) with the Need Satisfaction Scale, and occupational burnout symptoms with the Shirom-Melamed Burnout Measure. A hierarchical regression analysis and single slopes tests were performed to examine two- and three-way interactions.

**Results:** Stress was positively correlated with burnout, and negatively correlated with LTPA and PNS levels. LTPA was positively associated with PNS, and negatively correlated with burnout. A negative association existed between PNS and burnout. In the hierarchical regression analysis, all main effects, two- and three-way interactions were significant. People who engaged in more LTPA reported fewer burnout symptoms, if they reported high stress. However, the potential of LTPA to buffer stress was particularly evident in participants who reported low PNS.

**Conclusion:** If adult workers are exposed to elevated stress, they are particularly likely to show increased burnout levels if they report low LTPA in combination with low PNS, specifically a lack of autonomy, competence and relatedness.

## Introduction

Chronically high perceived stress constitutes a heavy burden on individuals’ health, by increasing their risk for non-communicable diseases (Bergmann et al., 2014), jeopardizing their mental health (Siegrist, 2008), and ultimately leading to premature death (Redmond et al., 2013). Furthermore, it is one of the key factors in the development and persistence of occupationalburnout (Glise et al., 2010). Previous research has shown that both leisure-time physical activity (LTPA) and the satisfaction of basic psychological needs are important health resources (Deci and Ryan, 2000; Biddle and Mutrie, 2006) which may facilitate successful coping with stress. Given these insights, the purpose of the present study was to examine, in a sample of Swiss adult workers, whether a combination of low LTPA and unsatisfied psychological needs is associated with increased burnout symptoms among participants who perceive their lives as being stressful, as compared to individuals who report low stress levels. This study addresses an important void in the literature, since little is known so far about whether the potential of LTPA to buffer stress is modulated by other personal and/or social factors.

In the present study, a special focus is placed on occupational burnout symptoms, because they have been considered as a major indicator of impaired well-being in working populations (Schaufeli and Bakker, 2004). Taking into consideration the basic tenets of the Conservation of Resources (COR) theory (Hobfoll and Shirom, 2000), Shirom et al. (2006) defined burnout as an individual’s feeling of being emotionally exhausted, physically fatigued, and cognitively worn-out. Using this definition of burnout, research has shown that burnout is associated with both physiological and psychological health outcomes. On the physiological side, higher burnout scores are related to increased cardiovascular risk factors such as increased fasting glucose, total cholesterol, low-density lipoprotein cholesterol and triglyceride levels (Gerber et al., 2016), increased cortisol levels throughout the day (Melamed et al., 1999), an elevated cortisol awakening response (Grossi et al., 2005), increased inflammation markers (Toker et al., 2005), and increased risk of developing type 2 diabetes (Melamed et al., 2006). As regards psychological dimensions, significant relationships exist between higher burnout symptoms, reduced life satisfaction and quality of sleep (Gerber et al., 2015), and depressive symptoms, with varying degrees of overlap (Schonfeld and Bianchi, 2016).

The relationship between LTPA and burnout symptoms is a relatively new area of scientific enquiry, because researchers have predominantly focused on depression as a mental health outcome (Mead et al., 2009). Nevertheless, evidence has increased during recent years, showing that a significant association exists between LTPA and burnout symptoms (Wunsch and Gerber, 2017). In a prospective study, Jonsdottir et al. (2010) showed that low LTPA predicted increased burnout symptoms over a 2-year period. In a cross-sectional study, Lindwall et al. (2012) found that lower self-reported LTPA levels were more closely associated with people’s burnout symptoms than objectively assessed cardiorespiratory fitness. Moreover, using latent growth curve analyses, Lindwall et al. (2014) showed in a longitudinal study that decreases in LTPA over a 4-year period are paralleled by an increase in burnout symptoms, and vice versa. In two cross-sectional investigations, Elliot et al. (2015) and Gerber et al. (2015) showed that vocational students who attain recommended levels of LTPA (according to internationally accepted standards) experience fewer burnout symptoms compared to peers with insufficient LTPA levels. Moreover, most of the existing experimental or quasi-experimental studies indicate that LTPA or exercise training leads to a reduction of burnout symptoms (Tsai et al., 2013; Bretland and Thorsteinsson, 2015; de Vries et al., 2017). From a stress-buffer perspective and based on cross-sectional data, Gerber et al. (2013b) reported a significant two-way interaction between self-reported stress and participants’ cardiorespiratory fitness levels. This indicates that the association between cardiorespiratory fitness and burnout symptoms is particularly strong in individuals who perceive high stress. Based on their findings, Gerber et al. (2013b) concluded that beyond primary prevention efforts to create less stressful work environments, promoting more active lifestyles could be a target for occupational health managers, because regular LTPA can strengthen employees’ capacity to cope with stress and stress-related disorders.

In summary, it appears that regular LTPA decreases the risk of developing burnout symptoms and can provide some protection against stressful life circumstances. However, the available research insufficiently accounts for the fact that other personal and social influences may serve as vulnerability factors or resilience resources (Luthar et al., 2006). In the present study, satisfaction with basic psychological needs is addressed for two reasons. First, previous research has indicated that personal factors might impact the ability of LTPA to buffer stress. For instance, focusing on the construct of hardiness, Kobasa et al. (1982) showed in a cross-sectional study that when exposed to high stress, participants with high hardiness and exercise scores remained more healthy than those with high scores in only one of these constructs. Similar findings were reported by Kobasa et al. (1985) in a prospective study showing that the total number of resistance resources (exercise, hardiness, and social support) predicted illness symptoms among participants exposed to high stress levels. Second, grounded in the self-determination framework (Deci and Ryan, 2000), previous investigations have pointed out that psychological need satisfaction (PNS) is positively associated with people’s well-being and contributes to their ability to deal with stress (Reis et al., 2000).

Self-determination theory (SDT) is an organismic meta-theory on human motivation and personality (Ryan and Deci, 2000; Deci and Ryan, 2002). Within this framework, the Basic Psychological Need Theory (BPNT) constitutes a mini-theory, which has become a popular approach to understanding how stressful life circumstances can lead to decreased performance and impaired well-being (Quested et al., 2011; Gunnell et al., 2013). A central tenet of BPNT is that satisfaction of basic psychological needs not only promotes human motivation and personality development, but is also key for well-being and the prevention of psychopathological symptoms. In this theory, Deci and Ryan (2000) define basic psychological needs as “those nutriments that must be procured by a living entity to maintain its growth, integrity and health” (p. 326). According to Van den Broeck et al. (2008), PNS is essential for humans to actualize their potential, to flourish, and to be protected from ill health and maladaptive functioning. There are three psychological needs in question, namely autonomy (e.g., having a choice and being able to determine one’s own actions), competence (e.g., feeling effective in interacting with one’s environment, feeling able to apply or develop one’s skills and capacities and to accomplish desired goals), and relatedness (e.g., feeling of mutual respect and having close relationships with other people, feeling love and care by significant others) (Vansteenkiste and Ryan, 2013). As mentioned above, prior studies support the notion that PNS is positively associated with psychological functioning across different life domains (Milayavskaya and Koestner, 2011). In the context of work, high PNS was found to be related to participants’ well-being (Lynch et al., 2005), job satisfaction (Ilardi et al., 1993), motivation (Gagné, 2003), dedication (Vansteenkiste et al., 2007), and performance (Baard et al., 2004), whereas low PNS was related to occupational burnout, both on an interpersonal (Vansteenkiste et al., 2007) and intrapersonal level (Aldrup et al., 2017). Furthermore, cross-sectional (Van den Broeck et al., 2008) and longitudinal evidence (Aldrup et al., 2017) suggests that PNS may function as a mediator between work-related stress and burnout symptoms. These studies indicate that stress may negatively affect PNS, which in turn may increase the risk of developing burnout symptoms. By contrasts, we are not aware of any previous studies in which PNS was conceptualized as a moderator, although such an approach accords well with a resilience perspective. As highlighted by Luthar et al. (2006), resilience researchers are generally interested in identifying vulnerability or protective factors that might modify the negative effects of adverse life circumstances (e.g., high subjective perception of stress) on health and well-being (cp. Masten, 2001). A protective factor is defined as something that moderates the effects of risk in a positive direction, whereas a vulnerability factor contributes to an exacerbation of the negative effects of risk. Resilience researchers assume that there are interindividual differences in protective and vulnerability factors, even though these factors also might be subject to change (and thus become mediators). Thus, in line with this perspective, one of the central objectives of our study is to find out whether the assumed (positive) relationship between stress and burnout symptoms is less pronounced among participants with higher levels of PNS compared to participants who report low PNS. To this end, we will test the three-way interaction between perceived stress, LTPA, and PNS on burnout symptoms.

**FIGURE 1:**
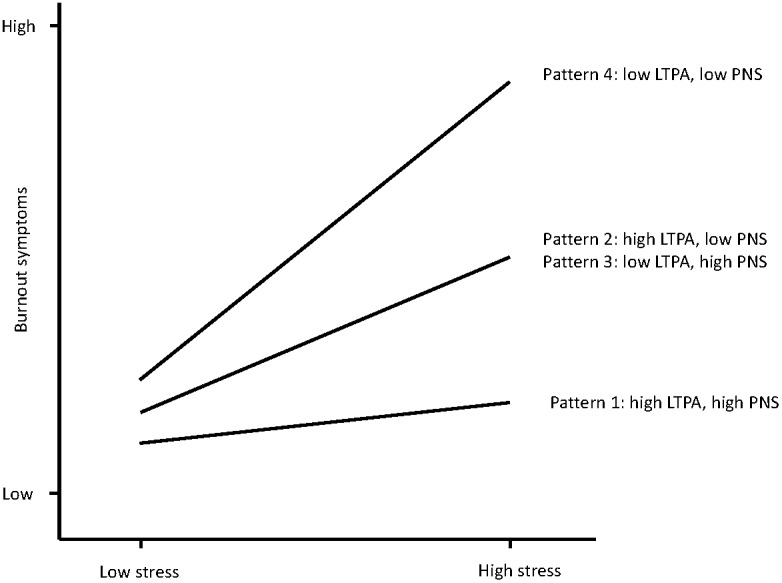
Conceptual model of the expected three-way interactions between stress, leisure-time physical activity (LTPA), and psychological need satisfaction (PNS) on burnout symptoms.

Four hypotheses were tested. First, we expected moderate-to-strong correlations between perceived stress, PNS (negative relationships) and occupational burnout symptoms (positive relationship) (Quested et al., 2011; Hämming et al., 2012; Aldrup et al., 2017). Second, we expected that LTPA would be positively associated with PNS (Wilson et al., 2006), and negatively associated with perceived stress (Stults-Kolehmainen and Sinha, 2014) and burnout symptoms (Jonsdottir et al., 2010). Third, we expected that LTPA and PNS would moderate the relationship between perceived stress and burnout, in the sense that if participants reported low stress levels, the relationship between LTPA or PNS and burnout symptoms would be relatively weak, whereas among participants with high stress levels, those with lower LTPA or PNS would report significantly higher burnout levels compared to participants with higher levels of LTPA and PNS. Our third hypothesis seems justified because the stress-buffering effects of LTPA are well documented (Klaperski, 2017). Similarly, previous studies have shown that variables associated with competence (Gerber et al., 2013a), autonomy (Bakker et al., 2005), and relatedness (Nakata et al., 2004) have the potential to protect against stress-related health complaints. Fourth, in line with previous research on stress, LTPA and hardiness (Kobasa et al., 1982), we expected that participants with low LTPA and PNS would experience the highest burnout levels if they are faced with stressful life circumstances. A conceptual model that illustrates the expected three-way interaction effects between stress, LTPA and PNS is depicted in Figure 1.

## Materials and Methods

### Participants and Procedures

Adult workers were recruited via undergraduate students (*N* = 87) who took part in an introductory course in research methodology at the University of Basel, Switzerland. Students were asked to provide contact details for six to twelve people (not relatives; 18–67 years old; ≥50% employment) who would be willing to take part in an online survey. Students identified 756 potential participants (46% women). The study was approved by the local ethics committee, and all participants gave written informed consent. After two reminders, 311 participants (161 men, 150 women; *M*_age_ = 42.64 years, *SD* = 14.20) filled in an online survey (41% response rate). All responding participants provided full data. Based on the Mahalanobis distance criterion, none of the participants had to be excluded as a multivariate outlier (based on stress, LTPA, PNS, and burnout scores). However, five participants were excluded as univariate outliers because their self-reported physical activity levels were +3 standard deviations above the mean. Therefore, the final sample (*N* = 306) used in the data analyses consisted of 159 men and 147 women, with a mean age of *M* = 42.93 years (*SD* = 14.12). Table 1 provides details regarding the social and demographic background of the sample.

**Table 1:** Social and demographic background of the study population.

Continuous variables	*M*	*SD*	Range
Age (in years)	42.93	14.12	19–67
Employment (past 3 months; % of full employment)	88.14	17.71	50–100
Years on job (in years)	21.82	13.94	1–47
Height	173.37	9.16	152–195
Weight	72.17	13.87	42–115
BMI	23.90	3.60	17.30–37.20

**Categorical variables**	***n***	**%**	

**Sex**			
Women	147	48	
Men	159	52	
**Marital status**			
Single	72	24	
In a relationship	234	76	
**Children living at home**			
Yes	125	41	
No	181	59	
**Responsibility as a caregiver**			
Yes	6	2	
No	300	98	
**Nocturnal shift work**			
Yes	26	8	
No	280	92	
**Highest completed education**			
Compulsory schooling	1	0	
Vocational education and training	89	29	
Commercial education or intermediate diploma school	40	13	
High school	28	9	
University or university of applied sciences	148	48	
**Smoking**			
Yes	45	15	
No	261	85	
**Use of psychotropic medication**			
Yes	4	1	
No	302	99	


### Measures

#### Perceived Stress

We used the German version (Klein et al., 2016) of the 4-item shortest version of the Perceived Stress Scale (PSS) (Cohen et al., 1983) to assess general perceived stress during the past month. The items measure the frequency with which respondents find their lives unpredictable, uncontrollable, and overwhelming (e.g., “How often have you felt that you could not control the important things in your life?”). A 5-point Likert scale ranging from 1 (never) to 5 (very often) is used as a response format. Items were summed to obtain an overall score. Validity and reliability of this instrument has been supported previously. While several studies reported acceptable psychometric properties of the PSS-4 (Cohen and Williamson, 1988; Leung et al., 2010), we acknowledged that the internal consistency and model fit was below recommended levels in some studies (Ingram et al., 2016). In the present sample, a confirmatory factor analysis showed that a 1-factor model achieved acceptable model fit, and that factor loadings were adequate (all loadings ≥0.45) (Supplementary Figure S1). Moreover, the Cronbach’s alpha was satisfactory (α = 0.75) in the present sample. Finally, previous studies showed that the associations of the PSS-4 with other stress- and health-related data were comparable with those of the PSS-14 and PSS-10 (Cohen and Williamson, 1988; Leung et al., 2010).

#### Leisure-Time Physical Activity

LTPA was measured with a German version (Mäder et al., 2006) of the short form of the International Physical Activity Questionnaire (IPAQ-SF) (Craig et al., 2003). A total index was built based on time spent in MPA (e.g., bicycling at a regular pace and low-intensity sports such as doubles tennis), and VPA (e.g., aerobics and fast bicycling), during the last week, using a frequency-by-duration format. Participants first indicated how many days per week they engaged in these activities (0–7 days), and then (if applicable) reported the average duration (in minutes). By multiplying frequency and duration scores, we obtained two weekly MPA and VPA estimates. Summing up these scores resulted in a total MVPA index. Adequate validity of the IPAQ has been reported previously (Craig et al., 2003).

#### Psychological Need Satisfaction

Satisfaction of the three core psychological needs was measured with the General Need Satisfaction Scale (GNSS) (Gagné, 2003). This instrument consists of 21 items which assess the extent to which the psychological needs of autonomy (7 items, e.g., “I feel like I can decide for myself how to live my life.”), competence (8 items, e.g., “I often do not feel very capable.”), and relatedness (6 items, e.g., “I really like the people I interact with.”) are satisfied. Three subscale scores were calculated by building the mean scores. Additionally, the three subscale scores were averaged to obtain a general PNS index. Because the GNSS subscales are highly correlated, we will only use the overall index when calculating two- and three-way interactions (Gagné, 2003; Vansteenkiste et al., 2007). However, descriptive statistics and bivariate correlations are reported for the overall PNS index and the subscales. Since this instrument was not available in German, we used a forward–backward translation procedure to create a German version of the GNSS, following the recommendations of Brislin (1986) (for German items see Supplementary Table S1). Evidence of the factorial validity is provided in the Supplementary Figure S2. As can be seen, the empirical data fitted adequately with the theoretical model. While the three latent factors were highly correlated with each other, all items loaded well on the expected factor (with one exception, all factor loadings ≥0.40). Cronbach’s alphas for the three subscales varied between 0.71 and 0.75, the Cronbach’s alpha for the overall PNS index was 0.86.

#### Occupational Burnout Symptoms

A German version (Gerber et al., 2018) of the 14-item Shirom-Melamed Burnout Measure (SMBM) (Melamed et al., 1999) was used to assess burnout symptoms. This instrument includes three subscales: (a) physical fatigue (e.g., “I feel physically exhausted.”), (b) emotional exhaustion (e.g., “I feel fed-up.”), and (c) cognitive weariness. Answers are given on a 7-point Likert scale, with response options ranging from 1 (almost never) to 7 (almost always). An overall SMBM index is derived by calculating the mean across all 14 items, with higher scores reflecting higher burnout symptoms. Sassi and Neveu (2010) showed that the SMBM has good psychometric properties, whereas Lundgren-Nilsson et al. (2012) found out that scores of ≥4.40 can be considered as clinically relevant. Similar to previous studies (Gerber et al., 2013b; Lindwall et al., 2014), the main analyses were only calculated for the overall burnout SMBM index to avoid the risk of alpha error inflation.

#### Confounders

The following social and demographic background variables were assessed as potential confounders, based on participants’ self-reports: age (in years), employment rate across the past 3 months (in % of full employment), years on job (in years), height (in m), weight (in kg), sex (0 = female, 1 = male), relationship status (0 = single, 1 = in a relationship), children at home (0 = no, 1 = yes), social responsibility toward relatives in need of care (0 = no responsibility, 1 = responsibility), nocturnal shift work (0 = no, 1 = yes), highest completed education, smoking (0 = no, 1 = yes), and use of psychotropic medication (0 = no, 1 = yes). Participants’ body mass index (BMI) was calculated with the following formula: kg/m^2^.

### Statistical Analyses

First, descriptive statistics (*M*, *SD*, range, skewness, and kurtosis) were calculated to describe the main study variables. Second, a series of analyses of variance (ANOVAs) and Pearson product moment correlations were calculated to find out whether participants’ burnout levels were associated with the potential confounders. Third, Pearson moment correlations were run to test bivariate relationships between the predictor (stress), moderator (LTPA and PNS) and outcome variables (burnout). Fourth, a χ^2^-test was performed to find out whether participants who met recommended LTPA levels were less likely to report clinically relevant burnout symptoms. Fifth, a hierarchical (five-stage) regression analysis was calculated to examine whether stress, LTPA, and PNS interacted in predicting burnout symptoms. In the first step, we controlled all potential confounders. In the second step, the main effect of stress was tested, followed by the examination of the main effects for LTPA and PNS in the third step. In the fourth step, all possible two-way interactions (stress^∗^LTPA, stress^∗^PNS, and LTPA^∗^PNS) were controlled. In the fifth step, the three-way interaction between stress, LTPA and PNS (stress^∗^LTPA^∗^PNS) was entered in the regression equation. All variables were z-standardized before the interaction terms were calculated. Moreover, as recommended by Dawson and Richter (2006), we used z-standardized values when performing the hierarchical regression analysis. The following statistical coefficients are provided in the results section: (i) the multiple correlation coefficient squared *R*^2^ and *F* value for the total model after step 5, (ii) the stepwise changes in *R*^2^ and *F*, and (iii) the standardized (β), unstandardized regression weights (B) and standard errors (S.E.) for each predictor variable (for the final model). In order to check for multicollinearity, variation inflation factor (VIF) was calculated. VIF scores ranged between 1 and 1.5 for all predictors, and were thus below critical levels (Kutner et al., 2004). Combined with the fact that all correlations between the predictor and moderator variables were below *r* = 0.50 (Donath et al., 2012), multicollinearity did not seem to be an issue in the present study. Significant two- and three-way interactions were plotted to facilitate interpretation of the results. To interpret the two- and three-way interactions, the results of the regression analysis plotted with high scores corresponding to values +1 *SD* and low scores to values –1 *SD*^1^. Moreover, for the three-way interactions, a simple slope analysis was performed to empirically test, which of the six possible pairs of slopes differ from each other (Dawson and Richter, 2006). The alpha probability level was set at *p* < 0.05 across all analyses. All analyses were calculated with SPSS^®^ (version 24, IBM Corporation, Armonk, NY, United States) for Apple Mac^®^.

## Results

### Descriptive Statistics

Table 2 displays the descriptive statistics for all study variables. In the present sample, 18 participants (5.9%) reported clinically relevant burnout symptoms (SMBM scores ≥4.40), whereas 99 participants (32.4%) did not attain LTPA recommendations ^2^, due to insufficient amounts of VPA (<75 min/week) and/or MVPA (<150 min/week).

**Table 2:** Descriptive statistics and psychometric properties of the major study variables.

				Range			
	*M*	*SD*	α (items)	Potential	Actual	Skewness	Kurtosis
Perceived stress	3.71	2.61	0.75 (4)	0–16	0–13	0.98	1.04
**Physical activity (min/week)**							
Overall LTPA (MPA + VPA)	230.19	183.73	— (4)	0+	0–840	1.06	0.88
MPA	98.57	87.65	— (2)	0+	0–600	1.63	3.53
VPA	132.41	148.83	— (2)	0+	0–840	1.79	3.73
**Psychological need satisfaction**							
Overall PNS index	5.46	0.61	0.86 (20)	1–7	2.82–6.94	–0.58	1.06
Autonomy	5.29	0.78	0.75 (7)	1–7	2.71–7.00	–0.63	0.39
Competence	5.50	0.75	0.72 (6)	1–7	2.67–7.00	–0.69	0.51
Relatedness	5.59	0.66	0.71 (7)	1–7	2.75–7.00	–0.43	0.81
**Burnout symptoms**							
Overall SMBM index	2.42	1.01	0.95 (14)	1–7	1.00–6.21	0.99	0.72
Physical exhaustion	2.72	1.26	0.92 (6)	1–7	1.00–7.00	0.88	0.35
Cognitive weariness	2.43	1.16	0.95 (5)	1–7	1.00–6.00	0.86	0.27
Emotional exhaustion	1.80	0.86	0.90 (3)	1–7	1.00–5.33	1.34	2.13


*LTPA, leisure-time physical activity; MPA, moderate physical activity; VPA, vigorous physical activity; PNS, psychological need satisfaction; SMBM, Shirom-Melamed Burnout Measure.*

### Socio-Demographic Background and Burnout Symptoms

Significant findings for burnout symptoms were as follows: Participants living in a relationship (*M* = 2.31, *SD* = 0.95) reported lower burnout scores, *F*(1,305) = 12.11, *p* = 0.001, η^2^ = 0.038, than singles (*M* = 2.78, *SD* = 1.10), whereas participants with children (*M* = 2.26, *SD* = 0.93) had lower burnout scores than those without children (*M* = 2.53, *SD* = 1.05), *F*(1,305) = 5.52, *p* = 0.019, η^2^ = 0.018. Moreover, those participants who take psychotropic medications reported higher burnout symptoms (*M* = 4.03, *SD* = 0.85) than participants who do not (*M* = 2.39, *SD* = 0.99), *F*(1,305) = 13.44, *p* = 0.001, η^2^ = 0.042. Finally, age, *r* = -0.27, *p* < 0.001, and years on job, *r* = -0.27, *p* < 0.001, were negatively correlated with self-reported burnout symptoms, highlighting that older and professionally more experienced participants reported lower burnout scores. Nevertheless, age and years on job were highly correlated with each other, *r* = 0.95, *p* < 0.001, and therefore we only used age as a covariate in the hierarchical regression analyses. No significant associations were found for the following variables: sex, *F*(1,305) = 0.23, *p* = 0.635, education, *F*(4,305) = 0.80, *p* = 0.526, employment, *F*(5,305) = 0.95, *p* = 0.447, caregiving, *F*(1,305) = 0.87, *p* = 0.350, nocturnal shift work, *F*(1,305) = 0.55, *p* = 0.460, smoking, *F*(1,305) = 0.23, *p* = 0.631, height, *r* = 0.045, *p* = 0.431, weight, *r* = 0.00, *p* = 0.961, and BMI, *r* = -0.03, *p* = 0.565.

### Bivariate Associations Between the Main Study Variables

As shown in Table 3, higher stress levels were strongly associated with more overall burnout symptoms (*r* = 0.56, *p* < 0.001). Participants with higher PNS reported lower stress perceptions (*r* = -0.49, *p* < 0.001) and fewer burnout symptoms (*r* = -0.51, *p* < 0.001). Similarly, participants with higher LTPA perceived less stress (*r* = -0.20, *p* = 0.001), indicated higher PNS (*r* = 0.30, *p* < 0.001), and reported fewer burnout symptoms (*r* = -0.37, *p* < 0.001).

**Table 3:** Summary of inter-correlations for cores on the main study variables.

	1	2	3	4	5	6	7	8	9	10	11
1. Perceived stress	—										
2. LTPA (MPA + VPA)	–**0.20^∗∗^**	—									
3. MPA	–0.10	0.55^∗∗∗^	—								
4. VPA	–0.14^∗^	0.86^∗∗∗^	0.19^∗∗^	—							
5. Overall PNS index	–**0.49^∗∗∗^**	**0.30^∗∗∗^**	0.17^∗∗^	0.26^∗∗∗^	—						
6. Autonomy	–0.46^∗∗∗^	0.25^∗∗∗^	0.15^∗^	0.20^∗∗^	0.88^∗∗∗^	—					
7. Competence	–0.51^∗∗∗^	0.30^∗∗∗^	0.19^∗∗^	0.24^∗∗∗^	0.84^∗∗∗^	0.63^∗∗∗^	—				
8. Relatedness	–0.23^∗∗∗^	0.21^∗∗∗^	0.09	0.20^∗∗∗^	0.77^∗∗∗^	0.54^∗∗∗^	0.43^∗∗∗^	—			
9. Overall SMBM index	**0.56^∗∗∗^**	–**0.37^∗∗∗^**	–0.20^∗∗∗^	–0.24^∗∗∗^	–**0.51^∗∗∗^**	–0.46^∗∗∗^	–0.55^∗∗∗^	–0.23^∗∗∗^	—		
10. Physical exhaustion	0.54^∗∗∗^	–0.27^∗∗∗^	–0.17^∗∗∗^	–0.21^∗∗∗^	–0.50^∗∗∗^	–0.46^∗∗∗^	–0.54^∗∗∗^	–0.22^∗∗∗^	0.92^∗∗∗^	—	
11. Cognitive weariness	0.50^∗∗∗^	–0.36^∗∗∗^	–0.16^∗∗^	–0.30^∗∗∗^	–0.49^∗∗∗^	–0.43^∗∗∗^	–0.52^∗∗∗^	–0.25^∗∗∗^	0.91^∗∗∗^	0.72^∗∗∗^	—
12. Emotional exhaustion	0.35^∗∗∗^	–0.27^∗∗∗^	–0.17^∗∗^	–0.21^∗∗∗^	–0.41^∗∗∗^	–0.35^∗∗∗^	–0.36^∗∗∗^	–0.30^∗∗∗^	0.71^∗∗∗^	0.51^∗∗∗^	0.62^∗∗∗^


*LTPA, leisure-time physical activity; MPA, moderate physical activity; VPA, vigorous physical activity; PNS, psychological need satisfaction; SMBM, Shirom-Melamed Burnout Measure; correlations between overall indices are highlighted in bold. ^∗^*p* < 0.05; ^∗∗^*p* < 0.01; ^∗∗∗^*p* < 0.001.*

A χ^2^-test showed that among those participants with clinically relevant burnout symptoms (SMBM ≥ 4.40), only 22% (*n* = 4) accomplished recommended LTPA levels. This percentage was considerably higher in the group with SMBM scores below the 4.40 threshold (71%, *n* = 203). Thus, participants with insufficient LTPA levels were statistically overrepresented in the group with clinically relevant burnout levels, χ^2^(1,306) = 18.03, *p* < 0.001, ϕ = 0.236.

### Physical Activity as a Moderator of the Stress-Burnout Relationship

**FIGURE 2:**
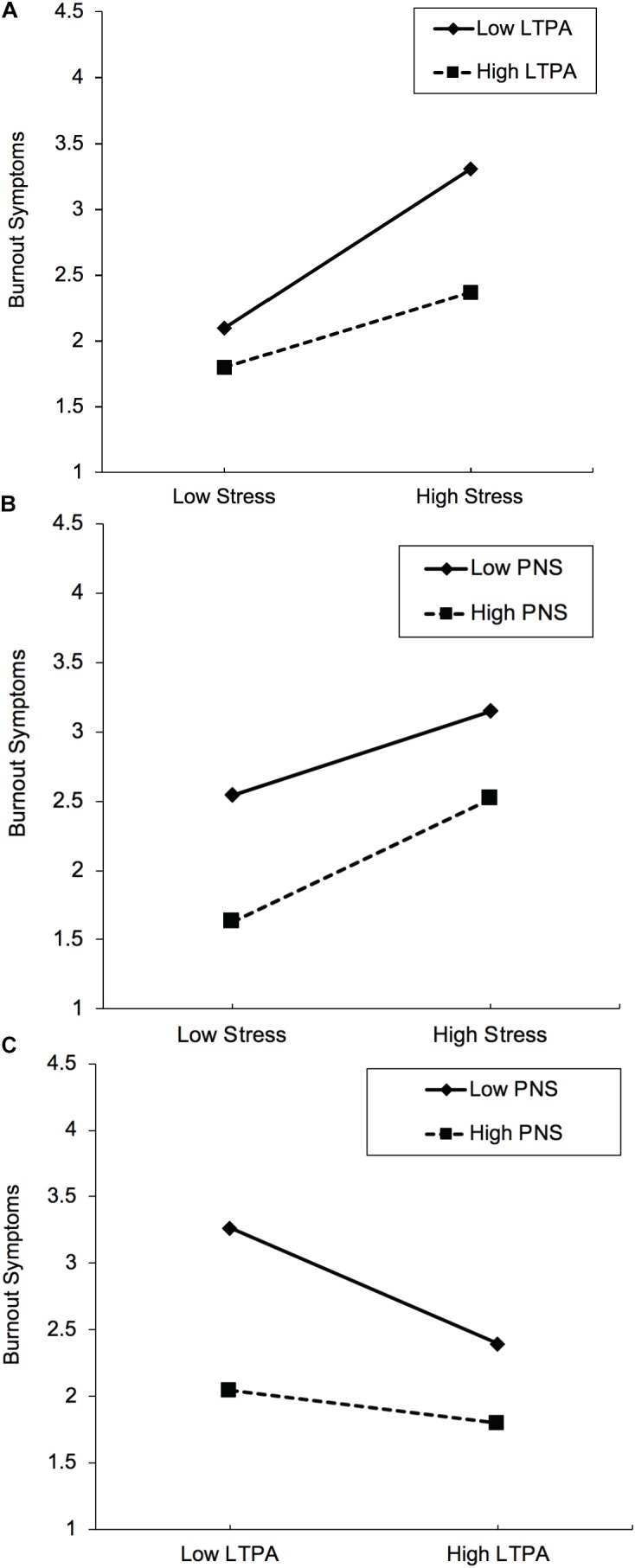
Two-way interactions between stress and leisure-time physical activity (LTPA) **(A)**, stress and psychological need satisfaction (PNS) **(B)**, and LTPA and PNS **(C)** on occupational burnout symptoms, after controlling for potential confounders.

**FIGURE 3:**
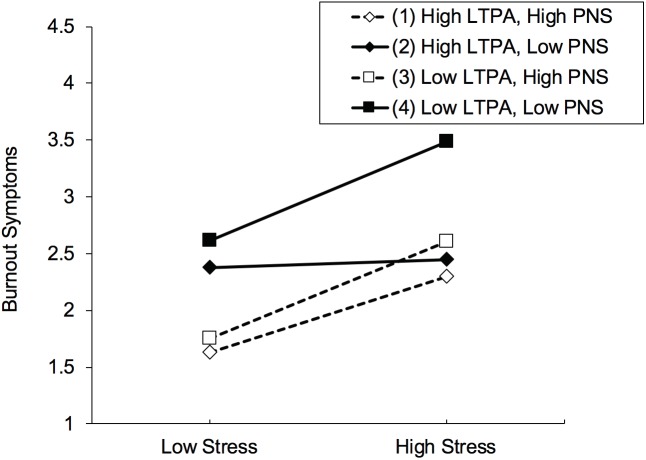
Three-way interaction between stress, leisure-time physical activity (LTPA), and psychological need satisfaction (PNS) on burnout symptoms, after controlling for potential confounders.

Table 4 contains the results of the hierarchical regression analysis. The overall model explained more than half (57.5%) of the variance in participants’ burnout symptoms. More specifically, in the first step, confounders explained 13.0% of variance, indicating that older participants, those living in a relationship and those not taking psychotropic medication reported fewer burnout symptoms. In the second step, perceived stress explained an additional 25.1% of variance in burnout (β = 0.30, *p* < 0.001), showing that higher stress is associated with more burnout symptoms. In the third step, higher LTPA (β = -0.21, *p* < 0.001) and PNS levels (β = -0.33, *p* < 0.001) were associated with fewer burnout symptoms (explaining 14.5% of additional variance). In the fourth step, the regression analysis yielded three significant two-way interactions between stress and LTPA (β = -0.12, *p* = 0.016), stress and PNS (β = 0.10, *p* = 0.029), and LTPA and PNS (β = 0.11, *p* = 0.020), explaining a further 4.2% of variance in burnout symptoms. Figure 2 provides insights regarding the direction of the two-way relationships. Thus, only small differences were found between participants with low vs. high LTPA if stress perceptions were low (Figure 2A) (note that in the plotted regression analyses, low vs. high values are defined as one standard deviation below versus above the mean). Nevertheless, when participants rated their lives as being stressful, a gap opened up, showing that participants with low LTPA report more burnout symptoms than participants with high LTPA. With regard to the interaction between stress and PNS (Figure 2B), the interaction pointed in a different direction. Thus, differences in burnout between participants with low vs. high PNS were bigger when participants reported low stress levels. The two-way interaction between LTPA and PNS reveals that participants with low LTPA and PNS levels report significantly more burnout than participants with low LTPA and high PNS (Figure 2C). Finally, in the fifth step, the three-way interaction between stress, LTPA and PNS explained 0.7% of variance in burnout symptoms (β = 0.10, *p* = 0.027). The most important finding of the plotted interaction (Figure 3) is that if the level of perceived stress is high, participants with low PNS and low LTPA report significantly higher burnout symptoms than all other participants. The simple slope tests further showed that three pairs of slopes significantly differed from each other (see Table 5). More specifically, the slope of those participants with high MVPA and low PNS levels (flat slope) differed from participants with (a) low MVPA/high PNS and (b) high MVPA/high PNS, in the sense that the latter two groups reported lower burnout levels if stress levels were low, whereas they exhibited similar burnout scores when stress levels were high. In contrast, participants with high MVPA and low PNS levels differed from their counterparts with low MVPA and low PNS, in the sense that the latter group had similar burnout symptoms if stress perceptions were low, whereas they had higher scores if they reported high stress levels.

**Table 4:** Hierarchical multiple regression analyses predicting burnout symptoms with age, relationship status, use of psychotropic medication, perceived stress, leisure-time physical activity and psychological need satisfaction.

	Δ*R*^2^	ΔF	β	SE
**Step 1**	0.130	4.40^∗∗∗^		
Age			–0.22^∗∗∗^	0.05
Employment rate			0.10^∗^	0.04
Marital status			0.00	0.05
BMI			0.03	0.04
Relationship status			–0.04	0.04
Children at home			0.01	0.05
Social responsibility toward relatives			0.01	0.04
Nocturnal shift work			0.09^∗^	0.04
Highest completed education			0.04	0.04
Smoking			–0.03	0.04
Use of psychotropic medication			–0.05	0.05
**Step 2**	0.251	119.07^∗∗∗^		
Stress			0.30^∗∗∗^	0.05
**Step 3**	0.145	44.79^∗∗∗^		
LTPA			–0.21^∗∗∗^	0.04
PNS			–0.33^∗∗∗^	0.05
**Step 4**	0.042	9.41^∗∗∗^		
Stress × LTPA			–0.12^∗∗^	0.05
Stress × PNS			0.10^∗^	0.03
LTPA × PNS			0.11^∗^	0.05
**Step 5**	0.007	4.95^∗^		
Stress × LTPA × PNS			0.10^∗^	0.04
Total *R*^2^	0.575			
Total *F*	311.56^∗∗∗^			
Constant	2.40			


*LTPA, leisure-time physical activity; PNS, psychological need satisfaction; regression weights are presented as they are after Step 5. ^∗∗∗^*p* < 0.001; ^∗∗^*p* < 0.01; ^∗^*p* < 0.05.*

**Table 5:** Slope difference test, comparing slopes depicted in Figure 3.

Pair of slopes	*t*-value for slope difference	*p*-value for slope difference
1 = high LTPA, high PNS		
2 = high LTPA, low PNS		
3 = low LTPA, high PNS		
4 = low LTPA, low PNS		
(1) and (2)	3.30	0.001
(1) and (3)	–0.71	0.481
(1) and (4)	–0.98	0.326
(2) and (3)	–3.07	0.002
(2) and (4)	–3.70	0.000
(3) and (4)	–0.09	0.928


## Discussion

The purpose of the present study was to examine the three-way interaction between perceived stress, LTPA, and PNS on occupational burnout symptoms. The key finding of the present study is that if adult workers experience high levels of stress, they are particularly likely to show increased burnout levels if they report low LTPA in combination with low PNS, defined as a lack of autonomy, competence and relatedness.

The present study adds to the current literature, as we examined for the first time whether the potential of LTPA to buffer stress-related health complaints depends on whether participants feel their basic psychological needs are (more or less) satisfied. The intention of this study was to explore new territory and give traditional stress-buffer research new impetus by linking LTPA with one of the most popular theories to explain human motivation and development. Thus, grounded in SDT (Ryan and Deci, 2000; Deci and Ryan, 2002), we were able to identify a specific target group for health interventions: individuals who combine low LTPA with low PNS.

In the present study, four hypotheses were tested. First, based on existing research (Quested et al., 2011; Hämming et al., 2012), we expected that stress would be moderately to strongly associated with low PNS and high burnout scores. This hypothesis was supported. Whereas stress is by definition seen as a key factor in the development, maintenance and exacerbation of burnout symptoms (Glise et al., 2010), SDT emphasizes that PNS greatly depends on the social context (Ryan and Deci, 2000). Thus, Vansteenkiste and Ryan (2013) argued that “whereas the satisfaction of the psychological needs for autonomy, competence, and relatedness contributes to proactivity, integration, and well-being, the frustration of these same psychological needs leaves one prone to passivity, fragmentation, and ill-being” (p. 263). In line with this notion, it is known from the literature that PNS mediates the relationship between self-perceived stress and burnout symptoms (Van den Broeck et al., 2008; Aldrup et al., 2017). The findings of our study show that measures on all levels (structural, social, and personal) seem worthwhile to reduce the volume of workers’ stress and their capacities to deal with challenging situations. Examples of such measures include family-friendly work environments (Secret and Sprang, 2002), creation of an autonomy-supportive climate at work (Havermans et al., 2017), or the promotion of workers’ stress management skills, e.g., via mindfulness-based approaches (de Vibe et al., 2012).

Our second hypothesis was that participants with higher LTPA would report elevated PNS. This hypothesis was supported, which corroborates previous investigations showing that LTPA can impact on participants’ well-being via increased PNS (Wilson et al., 2006; Gunnell et al., 2011). The findings of our study add to the current literature by showing that physically active participants generally see themselves as more autonomous, competent and socially involved. However, we acknowledge that our cross-sectional data cannot be interpreted causally. In other words, it cannot be ruled out that participants with lower PNS are just less motivated to engage in LTPA. Nevertheless, our study expands prior research in an important way, showing that LTPA is associated with general (domain-unspecific) PNS, whereas in sport and exercise psychology, researchers generally examined sport-specific PNS as predictor of sport and exercise behavior (Teixeira et al., 2012). Finally, our study points toward the potential of initiatives designed toward fostering physically active lifestyles among employees (Conn et al., 2009), not only to prevent chronic diseases (Kohl and Murray, 2012), but also to promote wellbeing and resilience resources (Gerber et al., 2012; Zayed et al., 2018).

Our third hypothesis was that LTPA and PNS would function as moderators of the stress-burnout relationship. In accordance with this assumption, we found that among participants with elevated stress levels, those with low LTPA reported significantly more burnout symptoms than those with high LTPA. This finding supports the potential of LTPA to buffer stress, which has been well documented in the extant literature (Klaperski, 2017). Both psychological, physiological and behavioral mechanisms have been suggested to explain why LTPA can moderate the relationship between stress and mental health, including differences in cognitive styles (Gerber et al., 2013a), sleep (Gerber et al., 2014a), health-related behaviors (Holmes, 2017), stress-reactivity (Hamer, 2012; Gerber et al., 2017), and cardiometabolic risk factors (Gerber et al., 2016). Furthermore, some researchers have argued that exercise and sport activities have the potential to facilitate recovery from stressful experiences (Sonnentag and Jelden, 2009). This is important because an efficient recovery has been identified as a relevant resilience factor in the work context (Sonnentag and Fritz, 2015). From a practical point of view and a burnout-prevention perspective, we may cautiously infer from our cross-sectional findings that regular physical activity is especially important/beneficial if a person is exposed to high stress levels (Naczenski et al., 2017). However, stress and a lack of time have been identified as central physical activity barriers (Stults-Kolehmainen and Sinha, 2014). Therefore, workplace physical activity programs should not only target physical activity behavior, but also behavioral skills that facilitate the implementation of sustainable behavior change (Michie et al., 2011; Nigg, 2013).

Our fourth hypothesis was also supported as stress, LTPA and PNS significantly interacted in the prediction of burnout symptoms. Among participants with high stress levels, those with low LTPA and PNS reported higher burnout levels than those with either high LTPA or high PNS. This finding is in line with previous research on hardiness (Kobasa et al., 1982, 1985), showing that individuals who lack resources across several domains (e.g., low LTPA levels in combination with low PNS) report lower well-being if they are exposed to stressful life circumstances. Conversely, if people perceive low stress levels, having high PNS may compensate for a lack of LTPA, especially in light of the fact that burnout levels were quite high in those with high LTPA and low PNS. Nevertheless, compared to previous studies on hardiness (Kobasa et al., 1982, 1985), no additive effect was found. Thus, if exposed to high stress, participants with high LTPA and high PNS levels reported similarly strong occupational burnout symptoms as counterparts who scored high in only one of these resources. Thus, our findings rather support a compensatory than an additive model. Taken together, we can state that among people with high stress levels, a lack of LTPA and PNS is associated with markedly higher burnout symptoms. Beyond that, our findings do not reveal whether LTPA or PNS should be considered as a more relevant health resource. Thus, when stress levels are low, having high PNS seems to be associated with lower burnout scores, independent of participants’ LTPA levels. However, when stress levels are high, a lack of LTPA in combination with low PNS seems to increase the risk of reporting higher burnout symptoms. Given this pattern of results, we can infer that fostering both resources (LTPA and PNS) is worthwhile, but their associations with wellbeing might depend on participants’ stress levels.

In order to avoid an overgeneralization of our findings, the following limitations should be considered regarding their interpretation. First, educational background was relatively high in the present study, which limits the generalizability of the data to groups with lower socio-economic status and from lower-status professions. Second, although comparable to other studies among Swiss workers (Gerber et al., 2010), only 40% of all invited participants completed the questionnaire. Third, the percentage of participants with clinically relevant burnout levels was lower than in previous studies (Gerber et al., 2014b), most likely due to the fact that students tended to approach more healthy individuals and those healthy individuals were more likely to complete the online survey. Fourth, the cross-sectional nature of this study does not allow a causal interpretation of both main and interaction effects. Fifth, interaction effects were only calculated for the composite PNS score. As highlighted by Quested et al. (2011) “this accounts for the tendency of the three needs to have shared variance, but precludes the possibility of determining the independent contribution of each need as a unique predictor of the outcome variables” (p. 832). Sixth, no information was available about the specific occupations participants were from (and response rates within each profession). However, we considered educational background when performing the regression analyses to account for the fact that differences might exist between white versus blue collar workers in their stress, LTPA, PNS and burnout symptoms. Seventh, the lack of objective measurement of LTPA is another limitation that should be addressed in future investigations. This is particularly true as researchers have expressed concerns about the validity of the IPAQ (Lee et al., 2011), because correlations between the IPAQ-SF and accelerometer data were of limited magnitude, and because the IPAQ-SF was associated with an overestimation of physical activity. Nevertheless, in a previous study with Swiss adults, the IPAQ-SF correlated reasonably well with accelerometer data (*r* = 0.43–0.50) (Mäder et al., 2006). Moreover, the IPAQ-SF was used successfully with Swiss adolescents to associate physical activity with burnout symptoms (Gerber et al., 2015). Finally, although estimation of physical activity via the IPAQ-SF might entail a certain measurement error, we were able to identify the expected relationships between physical activity and perceived stress (*r* = -0.20, *p* < 0.05), PNS (*r* = 0.30, *p* < 0.001) and burnout (*r* = -0.37, *p* < 0.001). Eighth, another direction for future research would be to examine how stress and PNS in different life domains, affect the relationships with occupational burnout. For example, does general PNS buffer work-related stress more than life related stress? Or are buffering effects stronger when needs are supported in the specific context in which there is a high source of stress (e.g., work stress + work PNS). Ninth, burnout is still not considered to be a distinct diagnosis in the ICD-10 (International Classification of Diseases) or DSM-5 (Diagnostic and Statistical Manual of Mental Disorders) classification systems. Thus, it would be interesting to know whether the observed moderation effects also occur for other mental health outcomes such as depressive symptoms. However, scholars are currently debating whether and to what degree burnout and depression can be considered as distinct constructs (Bianchi et al., 2015; Schonfeld and Bianchi, 2016). Tenth, we did not estimate sample size from previous studies examining the stress-buffer hypothesis. However, a *post hoc* power analysis showed that the final sample (*N* = 306) was large enough to detect (significant) bivariate correlations of *r* ≥ 0.14 (using G^∗^Power 3.1, point biserial model, one-tailed, α error probability = 0.05, Power = 0.80) between the study variables. Accordingly, our study was sufficiently powered to identify even small (main) effects of the predictor variables on the outcome. However, as scholars have highlighted previously (McClelland and Judd, 1993), detecting interaction effects with observational data is difficult because the interaction term is considered only after having accounted for the main effects (leaving limited variance to explain). Finally, the SMBM which was used in this study is not the same measure as SMBQ which was used in the Lundgren-Nilsson et al. (2012) paper which we referred to for clinical cut-offs. Although the items are similar, the cut-offs need to be interpreted with caution. Nevertheless, the ≥4.40 threshold is the only empirically derived cut-off, which we prefer to any arbitrarily set cut-off.

## Conclusion

Low levels of LTPA and low PNS were associated with more burnout symptoms and more perceived stress. Most importantly, participants who showed both low LTPA and low PNS when faced with stressful life circumstances reported the most burnout symptoms. Thus, based on our cross-sectional data, we cautiously conclude that participating in LTPA or feeling that one’s psychological needs (autonomy, competence, and relatedness) are satisfied may off-set some of the negative consequences associated with perceived stress, in particular symptoms of burnout. To counteract the burden associated with chronic stress exposure, we encourage the adherence to more physically active lifestyles and the creation of need-supportive climates in the family, in the leisure area, and at work.

## Availability of Data and Material

Can be requested for further analyses or transparency reasons from the corresponding author.

## Ethics Statement

This study was carried out in accordance with the recommendations of the Federal Act on Research involving Human Beings (Humanforschungsgesetz, HFG) with written informed consent from all subjects. All subjects gave written informed consent in accordance with the Declaration of Helsinki. The protocol was approved by the local ethical committee (EKNZ; Ethical Committee of Northwestern and Central Switzerland).

## Author Contributions

MG, SI-G, RS, SL, SB, and FC developed the study design, interpreted the data, revised the manuscript draft internally, and approved the final version of the manuscript. MG coordinated the study and was responsible for the data assessment. MG, SI-G, RS, SL, SB, and FC contributed to the statistical analyses. MG wrote the manuscript.

## Conflict of Interest Statement

The authors declare that the research was conducted in the absence of any commercial or financial relationships that could be construed as a potential conflict of interest.

## Footnotes

1 http://www.jeremydawson.co.uk/slopes.htm

2 www.cdc.gov/physicalactivity/basics/index.htm
