# Supplementary material for: When Low Leisure-Time Physical Activity Meets Unsatisfied Psychological Needs: Insights From a Stress-Buffer Perspective
Source: Front Psychol. 2018 Nov 2;9:2097. doi: 10.3389/fpsyg.2018.02097 (PMC6224427; doi:10.3389/fpsyg.2018.02097)
Supplement: Supplementary file 1 [file Data_Sheet_1.PDF]

Supplementary material

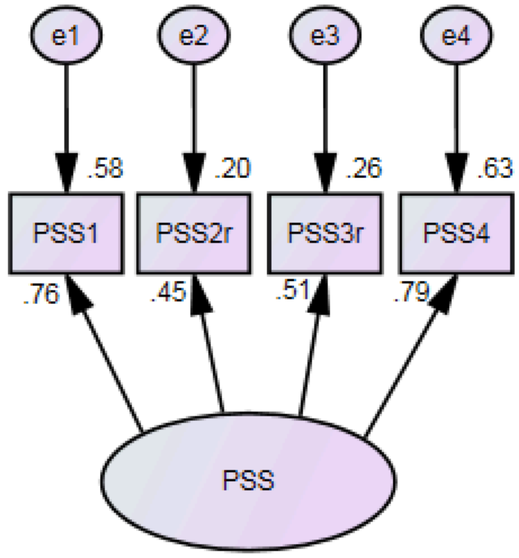

Figure 1S: Factorial structure of the German version of the PSS-4, used in the present study
Note. Model fit: RMR=0.02, TLI=0.90, CFI=0.98

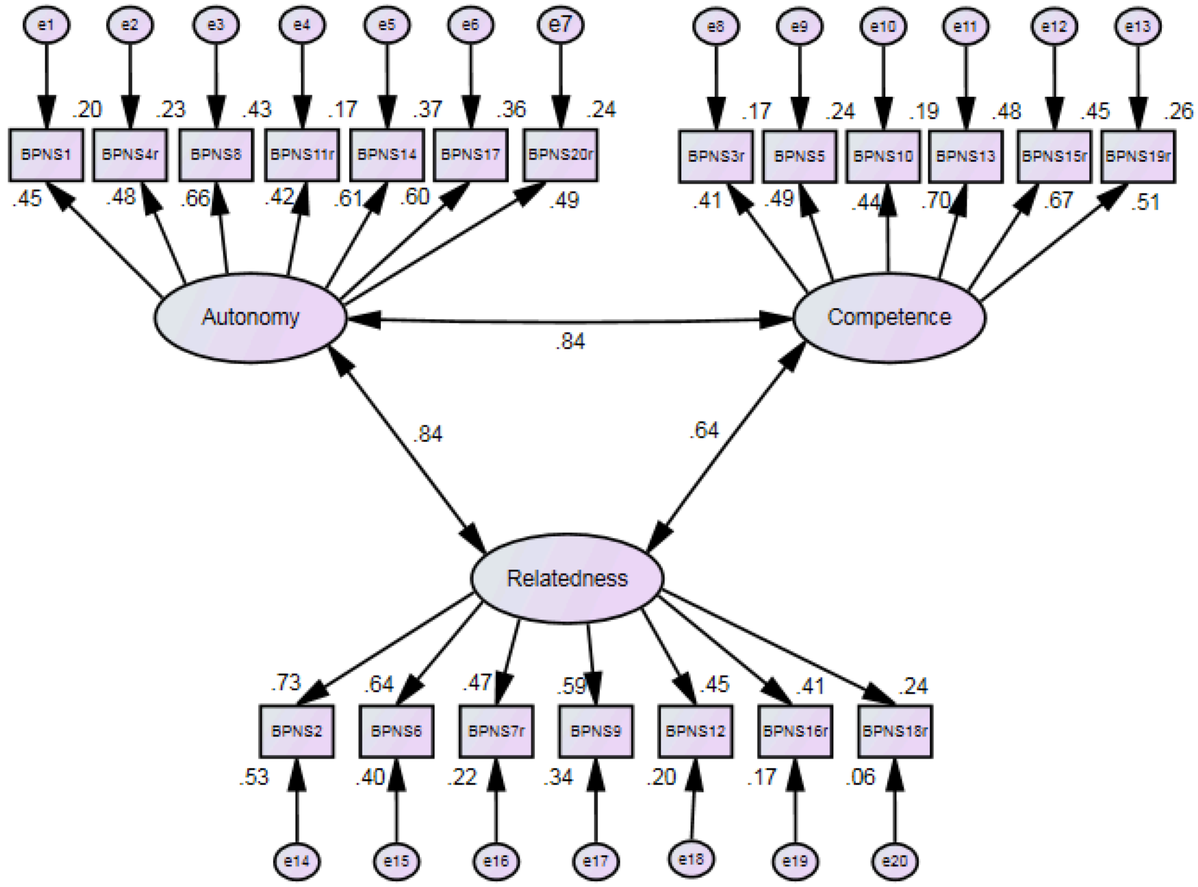

Figure 2S: Factorial structure of the German version of the GNSS, used in the present study
Note. Model fit: RMR=0.08, TLI=0.91, CFI=0.93

Table 1S. Wording of the German items of the General Need Satisfaction Scale (GNSS)

1	Ich fühle mich frei zu entscheiden, wie ich mein Leben gestalten möchte.
2	Ich mag die Leute sehr, mit denen ich zu tun habe.
3	Ich fühle mich häufig nicht besonders fähig.*
4	Ich fühle mich in meinem Leben häufig unter Druck gesetzt.*
5	Leute, die mich kennen, sagen mir oft, ich sei gut, in dem, was ich tue.
6	Ich verstehe mich mit den Leuten, zu denen ich Kontakt habe, meistens gut.
7	Ich bin eher ein Einzelgänger und habe nicht besonders viele soziale Kontakte.*
8	Es fühle mich normalerweise frei, meine Ideen und Meinungen auszudrücken.
9	Die Leute, mit denen ich regelmässig zu tun habe, zähle ich zu meinen Freunden.
10	Ich konnte kürzlich neue interessante Dinge zu lernen.
11	Im Alltag muss ich oft machen, was andere mir sagen.*
12	Die Menschen in meinem Leben kümmern sich um mich.
13	An den meisten Tagen empfinde ich durch das, was ich tue, ein Gefühl der Erfüllung.
14	Leute, mit denen ich täglich zu tun habe, nehmen Rücksicht auf meine Gefühle.
15	In meinem Leben kann ich nur selten zeigen, was ich wirklich drauf habe.*
16	Es gibt nicht viele Leute, die mir nahe stehen.*
17	Im Alltag kann ich meistens mich selber sein.
18	Die Menschen, mit denen ich regelmässig zu tun habe, scheinen mich nicht sehr zu mögen.*
19	Ich fühle mich oft inkompetent und ungenügend.*
20	In meinem Leben kann ich nur selten entscheiden, wie ich die Dinge gern hätte.*
21	Meistens sind die Leute mir gegenüber recht freundlich.

Notes. For the original items in English, go to http://selfdeterminationtheory.org/basic-psychological-needs-scale/. Answering options on the 7-point Likert scale: 1=Not at all true (Stimmt überhaupt nicht). 2=Not true (Stimmt nicht). 3=Rather not true (Stimmt eher nicht). 4=Neither nor (Weder noch). 5=Rather true (Stimmt eher). 6=True (Stimmt). 7=Completely true (Stimmt völlig)
*Items must be reverse-scored before calculating the mean (1→7, 2→6, 3→5, 4=4, 5→3, 6→2, 7→1)
